# Characterization of Two Historic Smallpox Specimens from a Czech Museum

**DOI:** 10.3390/v9080200

**Published:** 2017-07-27

**Authors:** Petr Pajer, Jiri Dresler, Hana Kabíckova, Libor Písa, Pavel Aganov, Karel Fucik, Daniel Elleder, Tomas Hron, Vitezslav Kuzelka, Petr Velemínsky, Jana Klimentova, Alena Fucikova, Jaroslav Pejchal, Rita Hrabakova, Vladimir Benes, Tobias Rausch, Pavel Dundr, Alexander Pilin, Radomir Cabala, Martin Hubalek, Jan Stríbrny, Markus H. Antwerpen, Hermann Meyer

**Affiliations:** 1 Military Health Institute, Military Medical Agency, Tychonova 1, 160 01 Prague 6, Czech Republic; pajer@img.cas.cz (P.P.); jiri.dresler@gmail.com (J.D.); hkabickova@centrum.cz (H.K.); l.pisa@email.cz (L.P.); agap@volny.cz (P.A.); fucik.karel@tiscali.cz (K.F.); 2 Institute of Molecular Genetics of the ASCR, v. v. i., Vídeňská 1083, 142 20 Prague 4, Czech Republic; daniel.elleder@img.cas.cz (D.E.); tomas.hron89@gmail.com (T.H.); 3 National Museum, Department of Anthropology, Václavské náměstí 68, 115 79 Praha 1, Czech Republic; vitezslav_kuzelka@nm.cz (V.K.); petr_veleminsky@nm.cz (P.V.); 4 Faculty of Military Health Sciences, University of Defence, Třebešská 1575, 500 01 Hradec Králové, Czech Republic; jana.klimentova@unob.cz (J.K.); alena.myslivcovafucikova@unob.cz (A.F.); jaroslav.pejchal@unob.cz (J.P.); 5 Laboratory of Applied Proteome Analyses, Institute of Animal Physiology and Genetics, Academy of Sciences of the Czech Republic, Rumburská 89, 277 21 Liběchov, Czech Republic; rita.hrabakova@gmail.com; 6 Genomics Core Facility, EMBL Heidelberg, Meyerhofstraße 1, 69117 Heidelberg, Germany; benes@embl.de (V.B.); rausch@embl.de (T.R.); 7 Institute of Pathology of the First Faculty of Medicine and General Teaching Hospital, Studničkova 2, 128 00 Prague, Czech Republic; pdundr@seznam.cz; 8 Institute of Forensic Medicine and Toxicology, First Faculty of Medicine, Charles University and General University Hospital in Prague, Studničkova 4, 128 21, Praha 2, Czech Republic; Alexander.Pilin@vfn.cz (A.P.); radomir.cabala@vfn.cz (R.C.); 9 Institute of Organic Chemistry and Biochemistry of the CAS, Flemingovo náměstí 542/2, 166 10 Praha 6, Czech Republic; martin.hubalek@uochb.cas.cz; 10 Military Institute of Forensic Medicine, Military University Hospital Prague, U Vojenské nemocnice 1200, 169 02 Praha 6, Czech Republic; jan.stribrny@uvn.cz; 11 Bundeswehr Institute of Microbiology, Neuherbergstr. 11, 80937 Munich, Germany; hermann1meyer@bundeswehr.org

**Keywords:** smallpox, variola virus, evolution, next generation sequencing, historic specimen, phylogeny

## Abstract

Although smallpox has been known for centuries, the oldest available variola virus strains were isolated in the early 1940s. At that time, large regions of the world were already smallpox-free. Therefore, genetic information of these strains can represent only the very last fraction of a long evolutionary process. Based on the genomes of 48 strains, two clades are differentiated: Clade 1 includes variants of variola major, and clade 2 includes West African and variola minor (Alastrim) strains. Recently, the genome of an almost 400-year-old Lithuanian mummy was determined, which fell basal to all currently sequenced strains of variola virus on phylogenetic trees. Here, we determined two complete variola virus genomes from human tissues kept in a museum in Prague dating back 60 and 160 years, respectively. Moreover, mass spectrometry-based proteomic, chemical, and microscopic examinations were performed. The 60-year-old specimen was most likely an importation from India, a country with endemic smallpox at that time. The genome of the 160-year-old specimen is related to clade 2 West African and variola minor strains. This sequence likely represents a new endemic European variant of variola virus circulating in the midst of the 19th century in Europe.

## 1. Introduction

Smallpox is caused by variola virus (VARV), which belongs to the genus *Orthopoxvirus* of the family *Poxviridae*. Smallpox is one of the most devastating diseases known to mankind. The characteristic skin rashes found on Egyptian mummies as well as ancient medical writings point to its emergence as early as 1100 to 1500 BC. Descriptions appear in 4th century AD in China, 7th century AD in India and the Mediterranean, and in the 10th century AD in southwestern Asia. By the mid-18th century, smallpox had spread all over the world [1]. It is believed that in the 20th century alone, 300 million people had been killed by smallpox [2]. In 1980, more than 200 years after the first vaccination campaign started, smallpox was declared eradicated. Today, World Health Organization (WHO)-sanctioned repositories of live variola virus are currently maintained only at two WHO collaborating centres: the Centers for Disease Control and Prevention, Atlanta, United States of America, and the State Research Center of Virology and Biotechnology VECTOR laboratory, Novosibirsk, the Russian Federation. However, smallpox still remains one of the most frightening biological threats because the majority of mankind is immunologically naïve today; any outbreak would be a major public health disaster of global proportions [3].

A set of 47 VARVs representing the greatest possible geographic and temporal diversity was selected from the WHO repository in Atlanta and sequenced [4]. Two primary clades emerged from sequence analysis. Clade P-I comprises Asian and African isolates; clade P-II comprises two subclades, one of them encompasses the biologically distinctive South American isolates known as Alastrim minor characterized by a markedly low fatality rate, and the other subclade is composed of strains isolated in West Africa. West African VARVs share a relatively recent common ancestor with Alastrim minor. Within the clades, VARVs cluster according to their geographical origin [4]. This is not surprising, since smallpox is transmitted via close human-to-human contact, which results in a rather slow spread. Epidemiological as well as genetic investigations clearly show strong evidence that all known European VARVs are linked to importations from Asia or Africa and thus do not represent true endemic “European” strains.

Since all of the 47 sequenced genomes were isolated between 1944 and 1977, which means a very short period on a historical scale, an unbiased estimate of time parameters for the molecular evolution of VARV is difficult to obtain. Research teams, using archival data on smallpox outbreaks combined with phylogenetic analyses, have obtained contradictory results on the possible time of VARV emergence [5,6,7] based on the method used. Shchelkunov and others [5,8] concluded that VARV most probably separated from a cowpox virus-like agent 3000–4000 years before present (YBP). Smallpox became endemic in West Africa in the 14th century AD and started to diverge in this geographically isolated region. Approximately 300–400 YBP, the West African VARV variant imported to South America started to evolve into the South American VARV Alastrim subtype.

In contrast, Li et al. [7] postulated that the two clades separated from the ancestral rodent virus either 16,000 or 68,000 YBP. Clade P-I spread from Asia either 400 or 1600 YBP. Clade P-II diverged from an ancestral VARV either 1400 or 6300 YBP and then diverged further into two subclades at least 800 YBP. Thus, the divergence of Alastrim and variola major occurred earlier than stated by Shchelkunov [9].

Recently a draft genome of VARV, sampled from a Lithuanian child mummy dating between 1643 and 1665, was reconstructed [10]. Strikingly, the mummy sequence fell basal to all currently sequenced strains of VARV on phylogenetic trees. Molecular-clock analyses revealed that the timescale of smallpox evolution is more recent than supposed, with the diversification of major viral lineages only occurring within the 18th and 19th centuries. Neither of the analyses explains from where the West African VARVs originate.

Several suspected smallpox specimens (scabs and entire corpses) that have surfaced since eradication have been investigated but failed to reveal VARV sequences useful for phylogenetic analysis [11,12,13,14,15]. Only very short sequences from a child (139 bp) and from a 300-year-old Siberian mummy (718 bp) have been amplified [11,15,16]. Here, we determined two complete VARV genomes from human tissues kept in a museum in Prague for 60 and 160 years, respectively. Our findings indicate that a VARV virus circulating in the midst of the 19th century in Europe has a common ancestor with West African and variola minor strains.

## 2. Materials and Methods

### 2.1. Diagnostic Specimens

**Figure 1:**
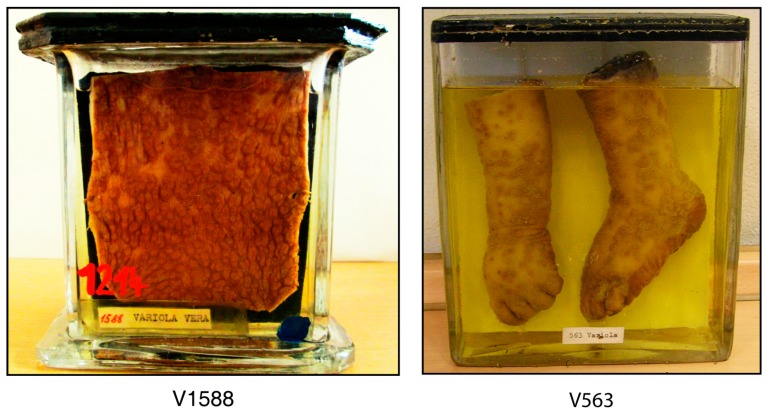
Anatomical specimens V1588 and V563 labeled “Variola”, Czech National Museum, Prague.

In the repository of the Czech National Museum (Prague) four preserved tissues, labeled “Variola” were discovered in 2014, but no documentation of the specimens’ history was available [17]. Sample V563 is composed of an intact forearm and a forefoot of a child, both clearly showing generalized exanthematous disease. Sample V1588 consists of a ca. 10 × 10 cm piece of skin, covered with numerous, discrete umbilicated pock lesions. Both specimens are visually highly suspicious of smallpox (Figure 1). In contrast, a third specimen, V1589, had been mislabeled since it seems to be a tumor of the skin and another specimen, AJ572, had been stored in a formaldehyde-based solution, attempts to extract genomic DNA completely failed. Therefore, specimens V1588 and V563 were chosen for further investigation.

### 2.2. Microscopy

Since the tissues had been immersed in a solution of unknown composition, sections of the specimens were washed several times in phosphate buffer saline (PBS) buffered formaldehyde before being processed for hematoxylin and eosin (H&E)-histology according to standard procedures. For electron microscopic inspections, small parts of the specimen were transferred into McDowell’s and Trump’s 4F:1G fixative (Sigma-Aldrich, Taufkirchen, Germany) for 19 days (3 changes) and rinsed in PBS pH 7.2 for 7 days (4 changes). Post fixation in 2% OsO_4_ (Polysciences, Hirschberg, Germany) in the same buffer for 2 h was performed. Subsequently, tissue specimens were dehydrated in a graded series of alcohol (30–100%), diluted with 5% aqueous solution of uranyl acetate (Polysciences), dehydrated in propylene oxide (Sigma-Aldrich) and embedded in resin Durcupan ACM (Sigma-Aldrich). Ultrathin sections (70 nm thick) obtained using diamond knives in a Reichert Ultra Cut microtome (Reichert, Vienna, Austria) were collected on copper grids (300-mesh) (Sigma-Aldrich). Grids were stained with 2% aqueous uranyl acetate and viewed at 80 kV with a microscope Jeol JEM, 2000 CX (Arishima, Tokyo, Japan) equipped with an Olympus Megaview II digital camera (Olympus Europa SE & Co, Hamburg, Germany).

### 2.3. DNA Extraction and PCR Analysis

Small pieces up to 3 mm in diameter of specimens V1588 and V563 were briefly washed in PBS and incubated in lysis buffer (0.25 M EDTA, 1% SDS, 1 mg/mL Proteinase K) for 24 h at 55 °C. Glycogen (20 µg) was added, and DNA was extracted by using phenol-chloroform. After ethanol-precipitation the DNA pellet was washed with 80% ethanol, dried and resuspended in 10 µL mM Tris-Cl/1 mM EDTA (pH 8.3). DNA was analyzed using Qubit 2.0 fluorometer (Thermo Fisher Scientific, Darmstadt, Germany) and a 2% agarose gel using standard protocols. One µL was used as template in two real-time PCR assays targeting sequences of the 14 kDa fusion protein gene and the cytokine response modifier B gene, respectively [18,19] and in a standard PCR assay targeting sequences of the hemagglutinin gene [20].

### 2.4. Age Estimation of Specimens and Composition of the Fixative Solution

In order to determine the age of the specimens, the degree of d-, l-aspartic acid racemization was analyzed as described previously [21]. Briefly, approximately 2 mg tissue was collected from each specimen and evaporated to dryness. A mixture of isopropanol and acetyl chloride (4:1, 100 µL) was added and heated to 100 °C for 30 min. The samples were evaporated to dryness under a stream of oxygen. A mixture of dichloromethane and trifluoroacetic acid anhydride (1:1, 100 µL) was added and heated to 60 °C for 15 min. The samples were again evaporated to dryness. The remaining material was dissolved in 100 µL of methyl acetate and analyzed by gas chromatography. The ratio of d- and l-aspartic acids was determined as the ratio of the respective peak areas. A mixture of d- and l-aspartic acids (1:10, Sigma) was used as a positive control. Ten tissue samples of known age from the National Museum depository were selected as calibration controls. The chemical composition of the fixative solutions of specimen V1588 and V563 was determined by gas chromatography and atomic absorption spectrometry (see Appendix A).

### 2.5. Next Generation Sequencing Analysis and Phylogenetic Analysis

DNA of specimens V1588 and V563 was subjected to Next Generation Sequencing (NGS) after DNA quality control using the Agilent Bioanalyzer (Agilent Technologies, Waldbronn, Germany). For sequencing, the HiSeq Illumina Plattform was chosen in combination with a 2 × 150 bp paired-end library using TrueSeq Nano DNA LT Sample preparation chemistry (Illumina, San Diego, CA, USA). Reads were background subtracted by mapping to the human chromosomes using BWA-MEM [22]. Reads not mapped were used for de novo assembly of contigs after “downsampling” to approximately 150×-coverage depth. Two different approaches, CLC Genomic Workbench (CLC) and software SPAdes [23] with the use of Pilon [24], were applied for error-correction. The BWA-MEM and Samtools-package [25] was used for variant calling compared to strain VARV-IND53_ndel (DQ441428). Consensus-sequences were extracted by both software-pipelines mentioned above, and differences were re-evaluated by manual inspection of the raw-assembly. For phylogenetic analyses, MAFFT-alignment [26] of all 48 available VARV virus genomes (Table S1) was generated and evaluated using MEGA 6.0 [27]. In addition, reads of the “mummy” genome VARV VD21 (PRJNA348754) were downloaded and processed equally to our samples as its genome sequence has not been made publically available. Annotation of the VARV strains was performed using GATU-utility [28].

### 2.6. Estimation of Molecular Clock

For estimation of the most recent common ancestors (MRCA) a highly conserved genome region, situated in the middle of the orthopoxvirus genome, was extracted for all strains. It comprises all genes between gene *F12L*, an actin-tail encoding gene, and *A32L*, an ATPase-DNA packaging protein. According to Upton et al. 2003 [29], this 104,142-bp-long region is known to be highly conserved in about 19 of 21 genomes. Using the BEAST Package [30], a Bayesian Markov Chain Monte Carlo (MCMC) method was used for estimation of the MRCA and its time of separation, as well as phylogenetic trees based on the calculated alignment. By using the Yule model for calculating a tree-prior, a constant speciation rate per lineage was assumed. In accordance to Babkin [5], this model was chosen, as it was calculated in their study to fit the best to this kind of selection and evaluation.

An uncorrelated relaxed clock model [31] combined with a Hasegawa-Kishino-Yano (HKY) substitution model [32] and a fixed tip date model was used. Tip dates of the year of isolation were set for strain VD21, V1588 and V563 to 1685 [10], 1850 and 1942, respectively. The latter dates were chosen in accordance with results of the d-, l-aspartic ratio for age estimation.

Twenty Mio steps of MCMC chains were run to ensure convergence, for which the initial 10% chains were used as burn-in and subsequently discarded. Using Tree Annotator of the BEAST package [30], the tree with the maximum sum of posterior probability after the burn-in experiments was chosen as a chronogram and the assumed time of separation for V563 and V1588 was determined.

### 2.7. High-Resolution Mass Spectrometry

The proteomes of the two historic specimens were determined by High-Resolution Mass Spectrometry. Because of potential interfering substances in the supernatant, a sample preparation workflow based on Filter Aided Sample Preparation (FASP) [33] was applied. Digested samples were analyzed via untargeted mass spectrometry on Q-Exactive (Thermo Fisher Scientific, Bremen, Germany) using a Nanospray Flex source (Thermo Fisher Scientific). The MS/MS spectral library was created by using Skyline software [34] and selecting the surrogate peptides including their heavy-labeled synthetic counterparts and transitions. Finally, all specimens were analyzed via targeted mass spectrometry employing 4000 QTRAP and 5500 QTRAP (AB Sciex, Darmstadt, Germany). A detailed description of the workflow is available in Supplementary Material and Methods S1.

## 3. Results

### 3.1. Diagnostic Specimens and Microscopy

**Figure 2:**
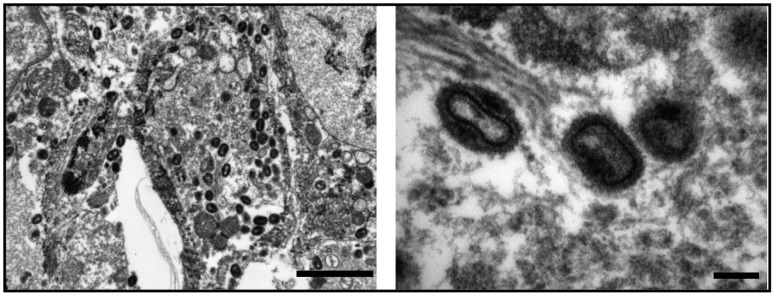
Electron microscopy of specimen V563 showing large numbers of typical orthopoxvirus particles at different magnification. Scale bars correspond to 2 micrometers (**left**) and 200 nm (**right**).

Two out of four specimens labeled “Variola” collected from the Czech National Museum in Prague showed an exanthematous rash with discrete and confluent lesions characteristic of smallpox (Figure 1). H&E-staining was suboptimal, presumably resulting from the age of the specimen, although morphological features, such as intraepidermal vesicle formation (pustules), ballooning of basal keratinocytes and karyorrhexis as well as intracytoplasmic inclusions and elementary bodies were found, which are consistent with a smallpox virus-induced cutaneous pock lesion (data not shown). Examination by electron microscopy showed large numbers of typical orthopoxvirus particles up to 250–300 nm in diameter (Figure 2).

### 3.2. Detection of Variola virus DNA

**Figure 3:**
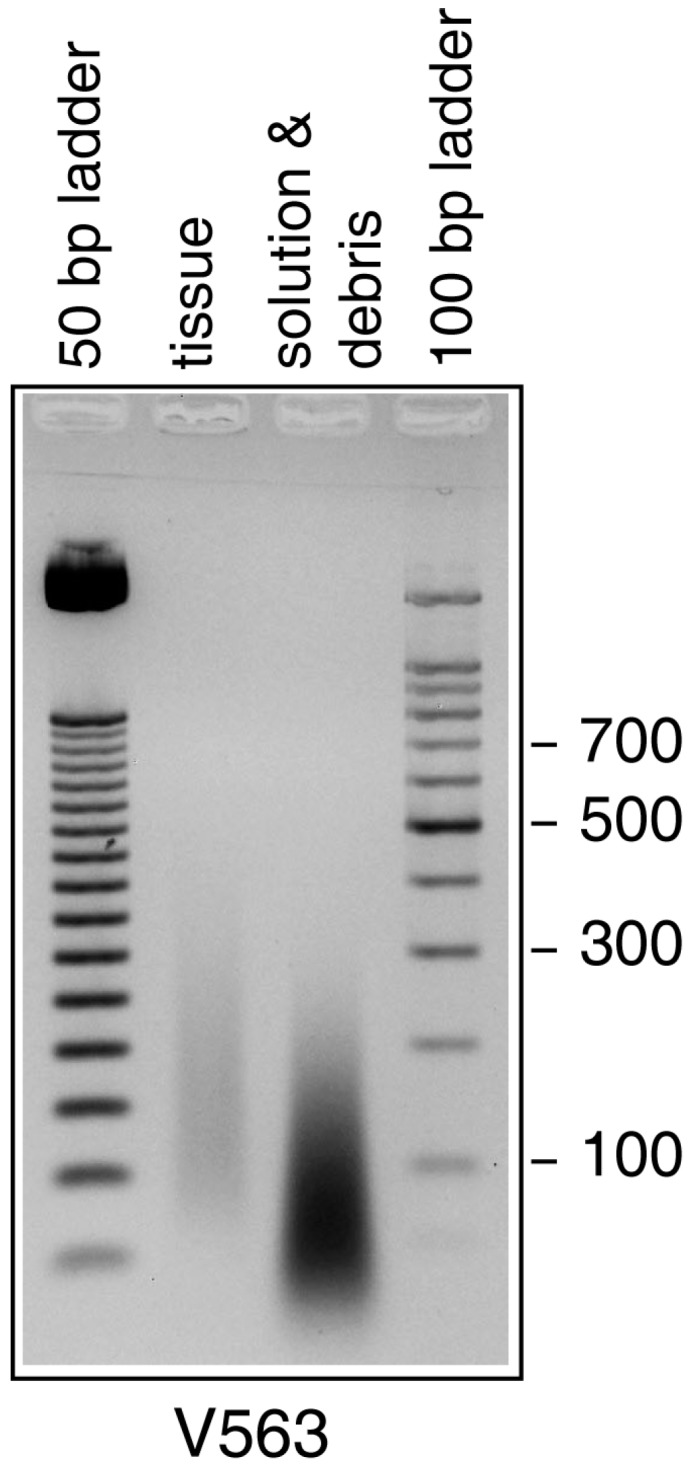
Agarose gel electrophoresis of ethidium bromide-stained DNA extracted from specimen V563.

DNA isolated from the specimens was highly fragmented and degraded, with an average fragment size of about 100–150 bp (V563) and about 200 bp (V1588), respectively (Figure 3). Two real-time PCR assays amplifying sequences of the *crmB* and the fusion protein gene yielded VARV-positive signals. Specificity was achieved by use of a VARV-specific probe [18] and by using hybridization probes followed by melting curve analysis [19]. Based on the C_t_-values, the number of VARV copies was determined to be about 25,000/assay. In contrast, an orthopoxvirus-specific PCR assay amplifying about 1000 bp of the hemagglutinin gene [20] was negative using template DNA of V563 or V1588, respectively (data not shown). 

### 3.3. Age of Specimens and Composition of the Fixative Solution

The age of specimens, as measured by the degree of Asp racemization, was estimated to be 62 ± 15 YBP for V563, which at 2 SDs calibrates to 1939–1969 AD. In the case of V1588, the age was determined to be 167 ± 40 YBP, which at 2 SDs calibrates to 1809–1889 AD, respectively. Results of the analysis including twenty specimens with known age used for calibration are summarized (Figure S2 and Table S2). The fixative/preservative solution, in which the specimens were kept, did not contain formaldehyde. In the case of V563, it was an aqueous solution with 2% sodium and 35% glycerol. In the case of V1588, it contained 1% sodium, 25% glycerol and 2.5% ethanol (Table S3).

### 3.4. High-Resolution Mass Spectrometry

**Figure 4:**
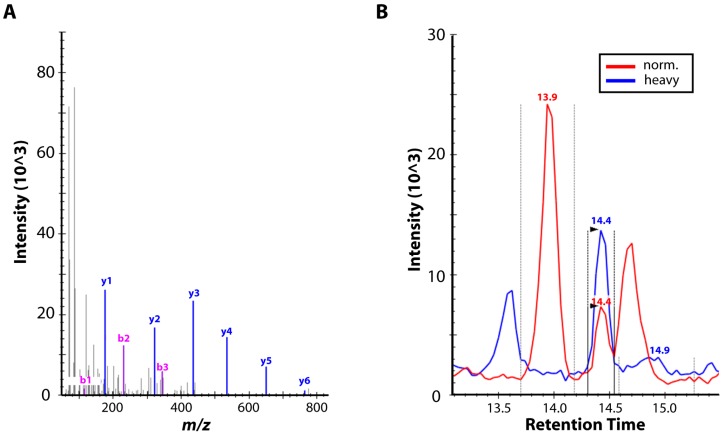
Mass spectrometric (MS) proteomic analysis. (**A**) Representative MS/MS spectrum of variola virus-specific peptide (NDDVLFR) from sample V1588. The spectrum depicts the intensity (*y*-axis) of analyzed mass-to-charge ratios of identified peptide fragments (*x*-axis). Based on this spectrum, fragments y4, y5 and y6 were involved in the Selected Reaction Monitoring (SRM) technique. (**B**) Selected Reaction Monitoring chromatogram of peptide (NDDVLFR) of sample V563. The peptide digest (in red) was analyzed with spiked synthetic, heavy-labeled counterpart (in blue). The presence of both peaks at a retention time of 14.4 min (based on prior heavy peptide analysis) together with the consistency of intensities of the ion pairs is an unambiguous evidence of the presence of peptide (NDDVLFR).

Using the obtained MS/MS spectral library, the targeted mass spectrometry method comprising 7 peptides of 7 different proteins (Table 1 and Table S3) was developed and re-applied onto specimens V563 and V1588. African Green Monkey Kidney cell line MA104 infected with cowpox virus strains EP-4 lidil and BR VR302, vaccinia virus strain Elstree B5, camelpox virus strain CP-1 and mock-infected MA104 cells were used as controls and analyzed in parallel. Based on mass spectrometry analysis, mock-infected MA104 cells did not contain any of the selected proteins/peptides, validating their reliability as a negative control. Extracts containing cowpox, vaccinia and camelpox virus infected specimens contained 6 out of the 7 peptides (except for the VARV-specific NDDVLFR peptide), proving their suitability as non-VARV-Orthopoxvirus materials. Conversely, specimens V563 and V1588 contained all 7 peptides (Figure 4). The sequence of peptide NDDVLFR, which is part of the 14 kDa fusion protein, is specific for VARV and can be used as a discriminatory marker.

### 3.5. Next Generation Sequencing and Phylogenetic Analysis

Sequencing of DNA of specimens V563 and V1588 on the Illumina platform generated around 400 and 87 million reads, respectively. Of these, 4.3% and 12.3%, respectively, were identified as poxvirus-specific. A virus consensus sequence was assembled for both specimens accounting for 183,535 bp (V563) and 184,287 bp (V1588) with a theoretical calculated average coverage depth of 1700-fold (V563) and 6900-fold (V1588) (Figure S3). The average Guanine-Cytosine (GC) content was calculated to be 33.3% (V563) and 32.8% (V1588). Both sequences contained all annotated genes found in the VARV reference sequence X69198 (Table S1). We identified and confirmed a total of 34 (V563) and 405 (V1588) single nucleotide polymorphisms (SNPs) relative to the VARV-IND reference sequence. Almost the entire genome was successfully sequenced, with the exception of the inverted terminal repeats at the outmost ends of the genome. Here, a reliable sequence could only be obtained for the last 75 and 79bp of NR2 (according to Massung et al. [35]) from V1588 and V563, respectively. The first open reading frame for both sequences is the *crmB* gene.

Sequences were submitted to the European Nucleotide Archive (ENA) and assigned accession numbers LT706528 (V563) and LT706529 (V1588). By Mauve alignment, the genomes revealed no major rearrangements compared to the variola major virus reference genome VARV-IND (DQ441427) and were strongly conserved in gene content and arrangement with all other VARVs isolated in the 20th century [4,36].

We investigated the gene *O1L* which is thought to be involved in virulence and host range specificity of members of the family *Poxviridae* [37]. Smithson et al. [37] compared SNPs specific for tatera poxvirus, camelpox virus and VARV versus SNPs specific for all other orthopoxviruses, such as cowpox, monkeypox and mousepox virus. In general, all SNP positions identified by Smithson et al. were also found and confirmed for samples V563 and V1588 and were identical to those SNP positions of other P1-clade VARV strains. In addition, strains VD21 and V1588 showed some additional SNPs: at position 1029 (a synonymous mutation) VD21 shows the non-variola “ATC” codon, whereas the codon “ATT” can be found in all other sequenced variola strains. This mutation may have occurred before division of the VARV lineages into P1 and P2-clades. At position 1705 one SNP (GAT → AAT, D → N) not mentioned by Smithson et al. [37] and specific for P2-clade strains including V1588 was observed.

**Figure 5:**
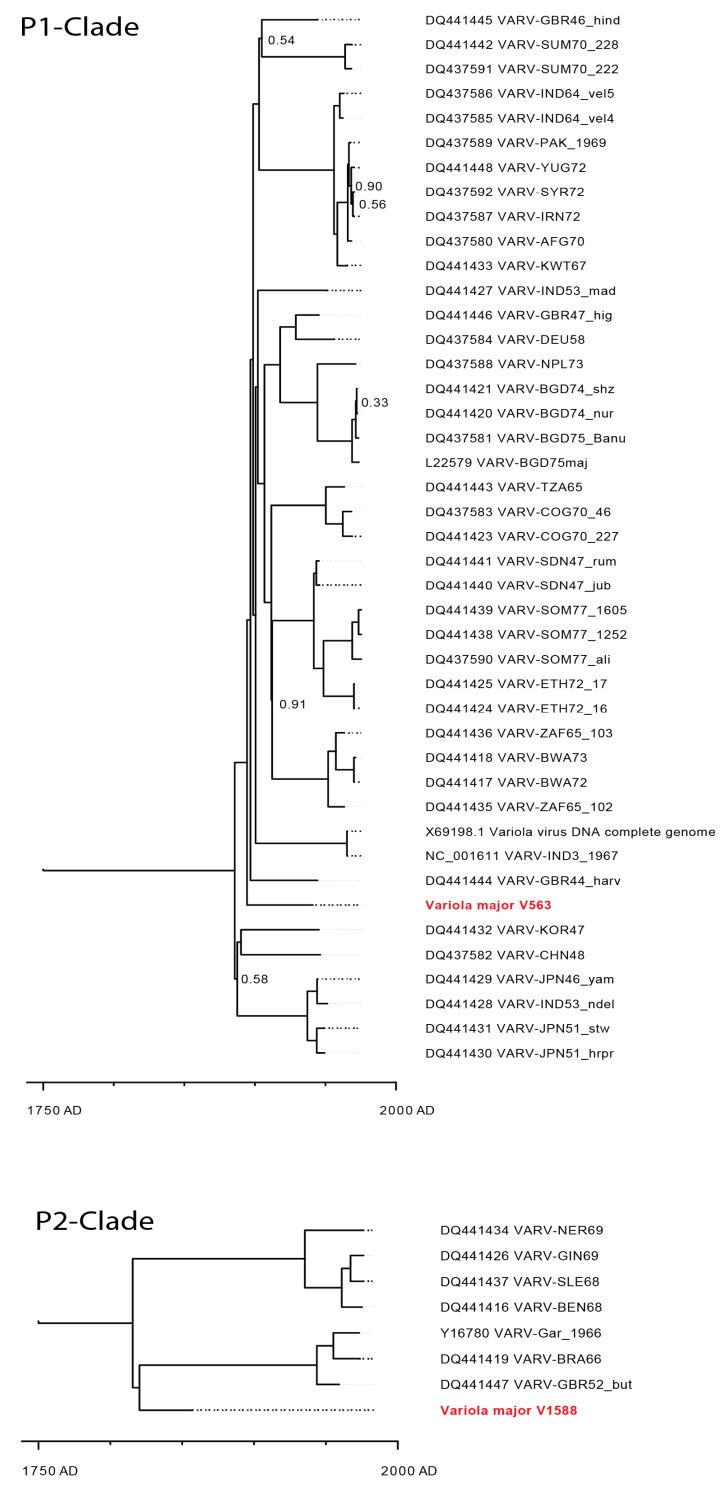
Maximum credibility tree for variola virus genomes, split into the major clades P-I and P-II. The chronogram was generated using BEAST software and is based on the highly conserved central genome region (104,142 bp). The posterior probabilities of all clades are >95% unless stated otherwise close to the nodes. Sequences of specimens V563 and V1588 are shown in red.

Phylogenetic analysis of V563 and V1588 with 48 complete genomes of VARV including a sequence derived from a 400-year-old Lithuanian mummy (Table S2) showed that V563 is a member of the P-I clade. It is most closely related to the VARV strains Harvey (DQ441444) and Hinden (DQ441445) differing in only 19 and 14 SNPs, respectively (Figure 5).

In order to obtain genetic information on the geographical origin of the human samples harboring V563 and V1588, the mitochondrial genomes for each sample were sequenced and their haplotypes determined. Both assigned haplotypes H1e (V563) and H1c (V1588) are common throughout Europe. Whereas H1e is found throughout Europe, H1c can be detected especially in Eastern Europe. These results are concordant with the assumption that these samples stemmed from Czech patients.

### 3.6. Molecular Clock Comparison

In contrast to the calculations reported by Duggan et al. [10], our molecular clock analysis indicates that VD21 shares a 200-year-older common ancestor with VARVs of the P1 and P2 clade dating around 1350 AD (Figure S3). This result was obtained by including the age of specimen V1588 (160 years) in the analysis. Separation within this 14th century led us to speculate that VD21 belonged to a separate and extinct lineage, but different from the already sequenced P1 and P2 strains of the 19th and 20th century (Figure S3). Uncertainty of this splitting date remains but may be decreased when additional historical samples with well documented sampling dates will be analyzed in the near future.

In contrast, phylogenetic analyses showed that V1588 has a rather unique sequence and is most closely related to the P-II clade; however, it could not be unambiguously assigned to either West African or variola minor subclade (Figure 5).

Modelling of the most recent common ancestor determined the divergence time of clades P-I and P-II at about 1695. With respect to our sample V1588, separation between the Alastrim- and the West African-Clade of P-II might have occurred around 1808 AD. Sample V563 showed a similar pattern to more recent P1-strains. However, the molecular clock analysis indicates that this specimen is rooted before the split of the most recent VARVs collected 1946 and later.

## 4. Discussion

The authors were fully aware that handling of variola virus DNA is governed by a series of recommendations made by the WHO Ad Hoc Committee on Orthopoxvirus Infections and by the WHO Advisory Committee on Variola Virus Research (ACVVR). Permission to perform the work reported herein was granted by ACVVR, and we analyzed these samples under maximum safety precautions and in accordance with locally agreed national guidelines. To rule out the persistence of intact viral particles, a long-distance PCR analysis (approximately 1 kb) was performed. No positive results were obtained, suggesting an extensive fragmentation of the viral genome, which was confirmed by gel electrophoresis with fragment lengths shorter than 200 bp that strongly support the presumption that the samples do not pose any further biological threat. After having conducted the experiments, all by-products containing variola virus DNA were disposed of by autoclaving. The characteristic skin lesions, the histological alterations, the demonstration of poxvirus particles and the amplification of VARV-specific DNA clearly proved that specimens V563 and V1588 had been obtained from patients infected with smallpox. Real-time PCRs yielded a rather high amount of VARV-specific DNA copies, whereas a standard PCR amplifying 1000 bp of the hemagglutinin gene was negative. This can be explained by degradation of DNA over time resulting in fragment sizes of 50 to 200 bp, which would allow efficient amplification of short amplicons in real-time PCR assays but not amplification of longer sequences. Since the size of the fragmented DNA (50 to 200 bp) was perfectly in a range needed for preparation of efficient libraries to perform next generation sequencing, a very high coverage (1700× and 6900×) was obtained subsequently, and two high-quality genomic sequences could be assembled. This success is mainly based on the fact that the fixative solutions of specimens V563 and V1588 did not contain formaldehyde, which usually causes extensive cross-linking and renders nucleic acids completely inaccessible [38,39]. In a previous study [15], analyzing a tissue preserved for decades in a solution containing formaldehyde, PCR yielded only very few copies of a short amplicon (139 bp) allowing no further differentiation of the VARV identified [15].

Furthermore, we demonstrated that a high-resolution mass spectrometry workflow can be a suitable complementary approach to the analysis of rare, barely accessible, or potentially dangerous agents such as VARV. More importantly, this approach could be used to detect VARV-specific peptides with great sensitivity in native biological material. While viable VARV-infected specimens are no longer accessible, the material stored in the Prague museum repository was invaluable in our efforts to test the performance of this alternative analytical method.

Phylogenetic analyses showed that V563 is a member of the P-I clade and that it is most closely related to VARV strains Hinden and Harvey. These strains were imported into the UK in 1946 and 1947 from the Indian subcontinent by infected travelers. At that time, smallpox was already eradicated from European countries. However, in the years between 1930 and 1946, a total of 68 cases of smallpox were observed in Czechoslovakia, all of which had been imported from endemic areas in India [3]. With an estimated age of 60 YBP, it is highly likely that V563 was one of the 68 cases that had occurred in Czechoslovakia during 1930 and 1946. Unfortunately, medical records of these smallpox cases do not exist anymore because archives were destroyed during World War II.

The age of V1588 was calibrated between 1800 and 1890, a period in which smallpox was endemic in European countries and had killed thousands of people especially after the German-French War in 1870/71. Phylogenetic analyses clearly assigned V1588 to the P-2 clade, which is divided into subclades consisting of variola minor and West African VARV strains. V1588 shares a common ancestor with both subclades, but branching is basal to the South-American or Alastrim clades. This ancestor could be a hitherto unknown European progenitor. The existence of a P-2 genotype in Europe is in line with phylogenetic analysis of the VARV sequence (VD21) of a 400-year-old Lithuanian mummy, which even fell more basal to the P-1 and P-2 clades [10]. It is tempting to speculate that American VARV strains are derived from VD21 due to past European exploration, colonization efforts and immigration. However, since specimens originating from the Americas are not available, it cannot be proven whether several older VARV lineages had been eradicated during the 19th and 20th century in the New World.

The relationship between the West African strains and a European progenitor suggests that smallpox could have been introduced into the densely populated areas of West Africa by European slave traders around 300–400 YBP. Infected sailors or traders returning from West Africa to continental Europe might have imported that lineage to Central Europe. Importation on this route in the midst of the 19th century might explain the smallpox epidemic into Central Europe, with a minor but still deadly virulent Alastrim-like lineage.

Within the gene *O1L*, the sequence of V1588 showed one nucleotide difference to V568 and VARV Bangladesh 1975 (DQ437581) at position 1705. This gene was investigated by Smithson et al. [37] as an evolution marker of host-range and therefore virulence. If the hypothesis of the significance of this SNPs for virulence is indeed linked to the case fatality rate, it could be argued that this SNP might be one responsible genetic determinant for a lower virulence or infection rate than that seen in variola virus major.

VARV isolates from South America and West Africa are distinct from each other but cluster in a common clade (P1-clade) considerably different from all other VARV isolates from Africa/Asia (P2-clade). Taking this into account, it is one mystery in the history of smallpox evolution to understand why VARV strains belong to either clade P1 or P2. It has been assumed that West African VARV variants were imported into South America and subsequently evolved into South American, i.e., Variola minor variants. Since genetic data of VARV strains are only available from the very last and rather short period prior to eradication, the history of smallpox evolution is a matter of speculation. And indeed, the existence of two clades and two subclades poses many questions: from where do West African VARV isolates originate? Are they related to European VARV variants? Is there a common, maybe a European progenitor of P1- and P2-clade viruses? What kind of VARV isolates have circulated in Europe, America and Russia? What is the relationship of those extinct agents with regard to the two clades differentiated today? Has smallpox repeatedly emerged from an ancestral zoonotic virus and disappeared because of insufficient population densities? All these questions can only be answered by analyzing historic specimens with new sequencing technologies available today and in combination with reliable methods for age estimation.

## Figures and Tables

**Table 1:** List of orthopoxvirus- and variola virus-specific proteins, peptides and their amino acid sequences.

Protein Name	Protein ID	Peptide	Specificity
VLFT-4 viral late transcription factor OS	Q0N822_VARV	ISAVSTVLEDVQAAGISR	*Orthopoxvirus*
ORF1L (Fragment)	Q89160_VARV	YQSLIPRLGINYLIDTTSR	*Orthopoxvirus*
Core protein VP8	VP8_VAR67	SmLSIFNIVPR	*Orthopoxvirus*
14 kDa protein	K7ZBW7_VARV	NDDVLFR	*Variola virus*
Cowpox A-type inclusion protein	Q0NBD3_VARV	VLLLTPEVASLR	*Orthopoxvirus*
Late transcription factor 1	VLTF1_VAR67	VNVFETR	*Orthopoxvirus*
Cowpox A-type inclusion protein	Q0NBD3_VARV	ISDLER	*Orthopoxvirus*

## Supplementary Materials

The following are available online at www.mdpi.com/1999-4915/9/8/200/s1, Figure S1: Linear regression model for estimation of the age of historic specimens based on the ratio of d-/l-Asp racemization; Figure S2: Schematic representation of the genomes derived from specimens V563 and V1588; Figure S3: Chronogram of the maximum credibility tree for Variola viruses generated using BEAST software based on the central conserved region of their genomes. Gray bars at nodes represent 95% highest probability density intervals. Numbers on nodes indicate the calculated year of the most recent common ancestor of the clades. The posterior probabilities of all clades is >95% except those labelled with a “#” next to the year; Table S1: Variola virus strains used for phylogenetic analyses; Table S2: Ratio of d-, l-aspartic acid racemization measurements; Table S3: Composition of the fixative solutions; Table S4: Overview of transitions for proteomic targeted qualitative analysis; Material and Methods S1.

## Appendix A. Analysis of the Preservative Solutions

Solutions in which specimens V563 and V1588 had been preserved, were analyzed for the presence of formaldehyde, glycerol and camphor by using a 6890N gas chromatograph (Agilent Technologies) with a 7683 Series autosampler (Agilent Technologies), a quadrupole mass spectrometer 5973 Inert (Agilent Technologies) and software MSD Chemstation Build 75 and Nist Mass Spectral Library v. 2.0 g. For analyses of Ag, Cd, Cr, Cu, Na, Ni and Pb the AA—240 FS Fast Sequential Atomic Absorption Spectrometer for flame AAS and the Varian AA—240 Z Zeeman Atomic Absorption Spectrometer with GTA Graphite Tube Atomizer (ETA AAS) were applied. The ethanol content was determined by gas chromatography employing a headspace technique on a GC-2010 Plus Gas Chromatograph with flame ionization detector (Shimadzu, Tokyo, Japan).
