# Supplementary figures and images for: Characterization of Two Historic Smallpox Specimens from a Czech Museum
Source: Viruses. 2017 Jul 27;9(8):200. doi: 10.3390/v9080200 (PMC5580457; doi:10.3390/v9080200)

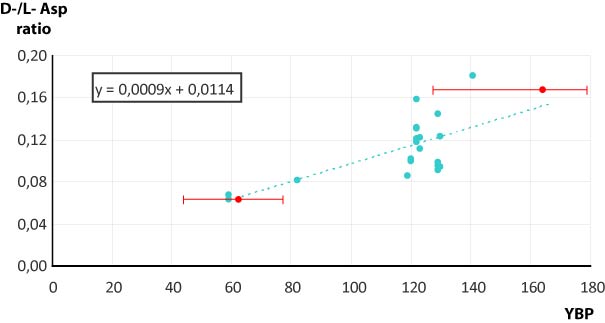

Supplement: Supplementary file 1 [file viruses-09-00200-s001.zip › manuscript historic smallpox/Supplementary Figure S1.jpg]

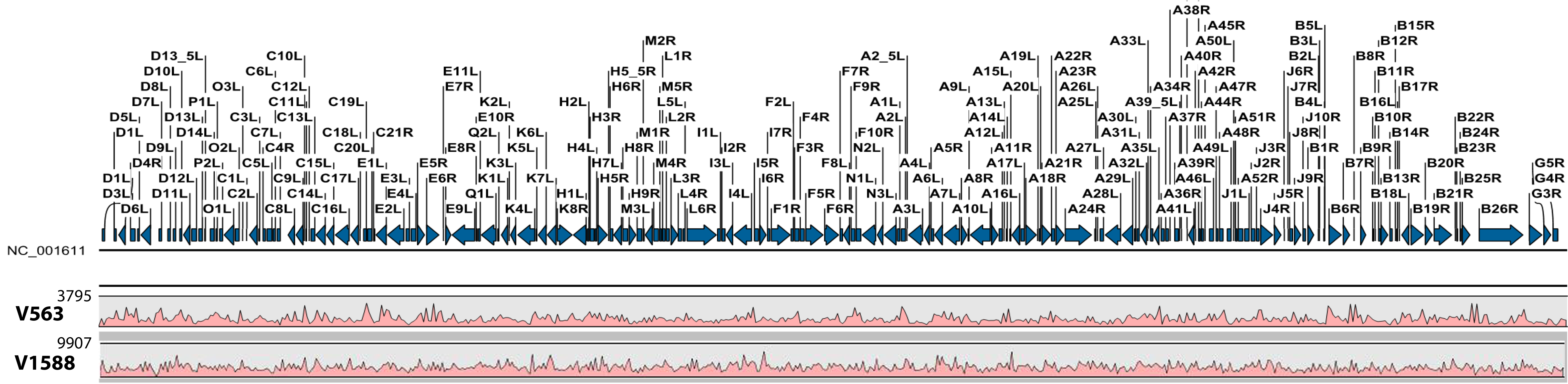

Supplement: Supplementary file 1 [file viruses-09-00200-s001.zip › manuscript historic smallpox/Supplementary Figure S2.tif]

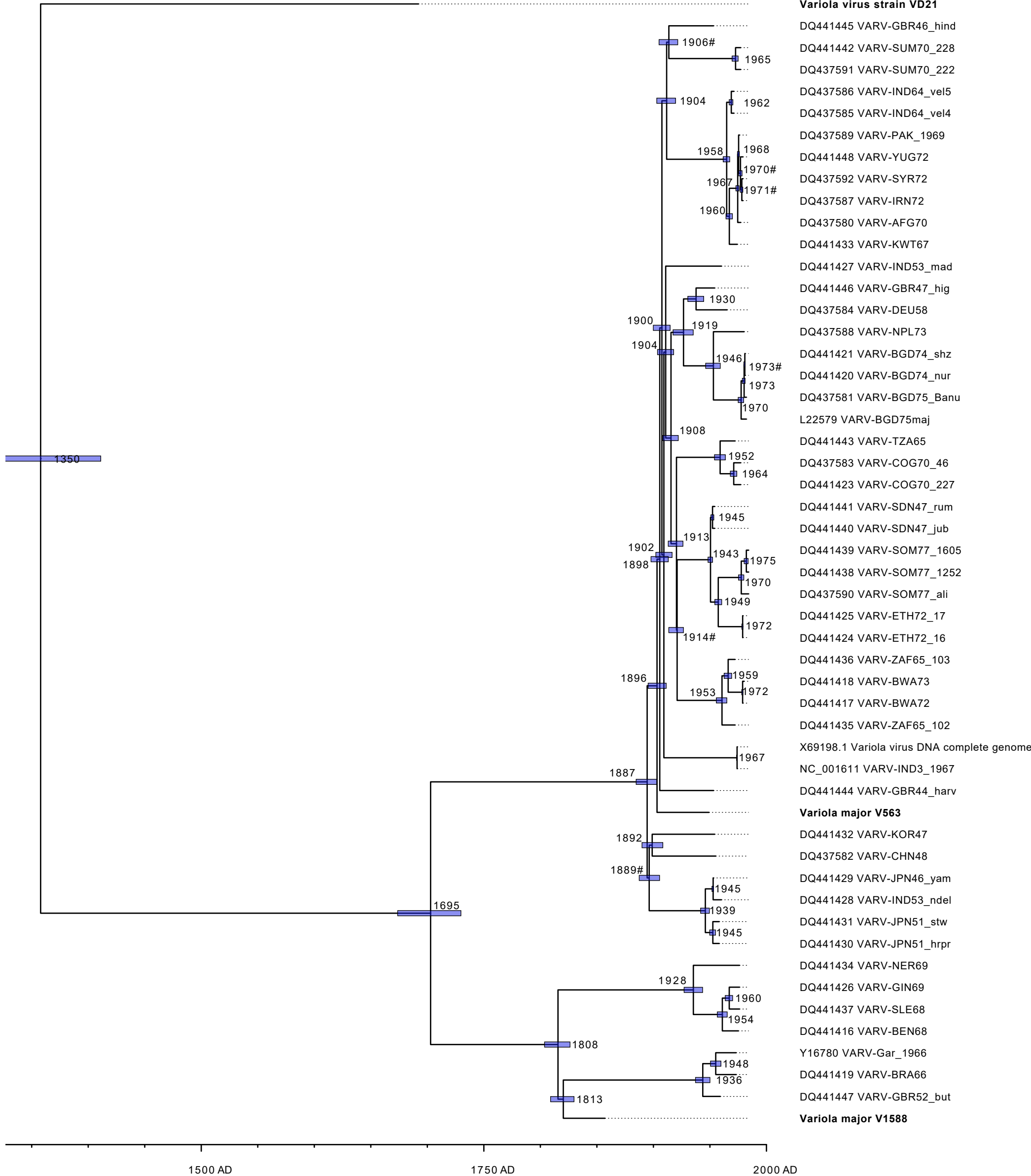

Supplement: Supplementary file 1 [file viruses-09-00200-s001.zip › manuscript historic smallpox/Supplementary Figure S3.pdf]
