# Supplementary material for: Characterization of Two Historic Smallpox Specimens from a Czech Museum
Source: Viruses. 2017 Jul 27;9(8):200. doi: 10.3390/v9080200 (PMC5580457; doi:10.3390/v9080200)
Supplement: Supplementary file 1 [file viruses-09-00200-s001.zip › manuscript historic smallpox/Variola_Manuscript supplement 20170626.doc]

**Supplementary Materials:** The following are available online at www.mdpi.com/link,

Figure S1: Linear regression model for estimation of the age of historic specimens based on the ratio of D-/L- Asp racemization

Figure S2: Schematic representation of the genomes derived from specimens V563 and V1588

Figure S3: Chronogram of the maximum credibility tree for Variola viruses generated using BEAST based on the central conserved region of their genomes. Gray bars at nodes represent 95% highest probability density intervals. Numbers on nodes indicate the calculated year of the most recent common ancestor of the clades. The posterior probabilities of all clades is >95% except those labelled with a “#” next to the year.

Table S1: Variola virus strains used for phylogenetic analyses

Table S2: Ratio of D-, L- aspartic acid racemization measurements

Table S3: Composition of the fixative solutions

Table S4: Overview of transitions for proteomic targeted qualitative analysis

Material and Methods S1

Table S1: Variola virus strains used for phylogenetic analyses

| **Accession number** | **Variola virus** | **Abbreviation** |
| --- | --- | --- |
| DQ435780 | VARV Afghanistan 1970 | VARV-AFG70 |
| DQ437581 | VARV Bangladesh 1975 v75-550 Banu | VARV-BGD75_Banu |
| DQ437582 | VARV China Horn 1948 Sabin Lab July 1948 | VARV-CHN48 |
| DQ437583 | VARV Congo 1970 v70-46 Kinshasa | VARV-COG70_46 |
| DQ437584 | VARV Germany 1958 Heidelberg | VARV-DEU58 |
| DQ437585 | VARV India 1964 7124 Vellore | VARV-IND64_vel4 |
| DQ437586 | VARV, India 1964 Velore5 | VARV-IND64_vel5 |
| DQ437587 | VARV Iran 1972 2602 Tabriz | VARV-IRN72 |
| DQ437588 | VARV Nepal 1973 V73-175 | VARV-NPL73 |
| DQ437589 | VARV Pakistan 1969 (Rafig Lahore) | VARV-PAK_1969 |
| DQ437590 | VARV Somalia 1977 V77-2479 | VARV-SOM77_ali |
| DQ437591 | VARV Sumatra 1970 V70-222 | VARV-SUM70_222 |
| DQ437592 | VARV Syria 1972 V72-199 | VARV-SYR72 |
| DQ441416 | VARV Benin Dahomey 1968 (v68-59) | VARV-BEN68 |
| DQ441417 | VARV Botswana 1972 (v72-143) | VARV-BWA72 |
| DQ441418 | VARV, Botswana 1973 | VARV-BWA73 |
| DQ441419 | VARV Brazil 1966 (v66-39 Sao Paulo) | VARV-BRA66 |
| DQ441420 | VARV, Bangladesh 1974, Nurislam | VARV-BGD74_nur |
| DQ441421 | VARV, Bangladesh 1974, Shahzaman | VARV-BGD74_shz |
| DQ441423 | VARV, Congo 1970, 9-V74-227Gispen | VARV-COG70_227 |
| DQ441424 | VARV Ethiopia 1972 (Eth16 R14-1X-72 Addis) | VARV-ETH72_16 |
| DQ441425 | VARV Ethiopia 1972 (17R14-1X72) | VARV_ETH72_17 |
| DQ441426 | VARV Guinea 1969 (005) | VARV-GIN69 |
| DQ441427 | VARV India 1953 (Kali-Muthu-M50 Madras) | VARV-IND53_mad |
| DQ441428 | VARV India 1953 (New Delhi) | VARV-IND53_ndel |
| DQ441429 | VARV Japan 1946 (Yamada MS-2(A) Tokyo) | VARV-JPN46_yam |
| DQ441430 | VARV, Japan 1951, Harper-Masterseed | VARV-JPN51_hrpr |
| DQ441431 | VARV, Japan 1951, Stillwell-Masterseed | VARV-JPN51_stw |
| DQ441432 | VARV Korea 1947 (Lee Masterseed) | VARV-KOR47 |
| DQ441433 | VARV Kuwait 1967 (K1629) | VARV-KWT67 |
| DQ441434 | VARV, Niger 1969 | VARV-NER69 |
| DQ441435 | Variola virus strain South Africa 1965 (102 Natal Ingwavuma) | VARV-ZAF65_102 |
| DQ441436 | Variola virus strain South Africa 1965, 103 Transvaal Nelspruit | VARV-ZAF65_103 |
| DQ441437 | VARV, Sierra Leone 1968, V68-258 | VARV-SLE68 |
| DQ441438 | VARV, Somalia 1977 | VARV-SOM77_1252 |
| DQ441439 | VARV, Somalia 1977 | VARV-SOM77_1605 |
| DQ441440 | VARV Sudan 1947 (Juba) | VARV-SDN47_jub |
| DQ441441 | VARV, Sudan 1947 | VARV-SDN47_rum |
| DQ441442 | VARV, Sumatra 1970, V70-228 | VARV-SUM70_226 |
| DQ441443 | VARV Tanzania 1965 kembula | VARV-TZA65 |
| DQ441444 | VARV United Kingdom 1946 Harvey | VARV-GBR44_harv |
| DQ441445 | VARV United Kingdom 1946 Hinden (Middlesex) | VARV-GBR46_hind |
| DQ441446 | VARV United Kingdom 1947 Higgins (Staffordshire) | VARV-GBR47_hig |
| DQ441447 | VARV United Kingdom 1952 Butler | VARV-GBR52_but |
| DQ441448 | Variola virus strain Yugoslavia 1972 V72-164 | VARV-YUG72 |
| L22579 | VARV, Bangladesh 1975, Variola major virus | VARV-BGD75maj |
| **LT706528** | **VARV V563, this paper** | **VARV-V563** |
| **LT706529** | **VARV V1588, this paper** | **VARV V1588** |
| NC_001611, X69198.1 | VARV, India 1967 | VARV-IND2_1967 |
| PRJNA348754 | VARV Lithuania, 17th century | VARV VD21 |
| Y16780 | VARV strain Garcia 1966 | VARV-Gar_1966 |

Table S2: Ratio of D-, L- aspartic acid racemization of specimens V563 and V1588 as compared to ratios of specimens with known age

| **Specimen** | **Years Before Present** | **Year** | **D-/L- Asp ratio** |
| --- | --- | --- | --- |
| V563 | 62 | 1954 +/- 15 | 0,062205592 |
| V1588 | 167 | 1849 +/- 40 | 0,167076973 |
| AJ130 | 141 | 1875 | 0,180403341 |
| 3622 sin | 120 | 1896 | 0,099014681 |
| 3622 dx | 120 | 1896 | 0,101463341 |
| 3673 DK dx | 122 | 1894 | 0,158121848 |
| 1886 DK sin | 130 | 1886 | 0,094247218 |
| 1886 DK dx | 130 | 1886 | 0,122659556 |
| 3610 DK sin | 123 | 1893 | 0,122307548 |
| 3610 DK dx | 123 | 1893 | 0,111359504 |
| 3611 DK sin | 129 | 1887 | 0,095119061 |
| 3611 HK sin | 129 | 1887 | 0,098478408 |
| 3611 HK dx | 129 | 1887 | 0,090871159 |
| 3611 HK dx | 129 | 1887 | 0,144409274 |
| 3673 HK dx | 122 | 1894 | 0,117404879 |
| 3673 HK dx | 122 | 1894 | 0,130422001 |
| 3673 HK sin | 122 | 1894 | 0,120613900 |
| 3673 DK sin | 122 | 1894 | 0,132008927 |
| 5078 Ach sin | 59 | 1955 | 0,063118615 |
| 5078 Ach dx | 59 | 1955 | 0,067485687 |
| 3674 DN | 82 | 1934 | 0,081015444 |
| 3795 DR | 119 | 1897 | 0,086053644 |

Table S3: Composition of the fixative solutions

| **Component** | **Fixative solution of V563** | **Fixative solution of V1588** |
| --- | --- | --- |
| Glycerol | 35% | 25% |
| Sodium (Na), g/l | 20.9 | 10.2 |
| Ethanol (m/m%) | 0.02% | 2.5% |
| Formaldehyde (m/m%) | 0 | 0 |
| Copper (Cu), µg/l | 776 | 1552 |
| Nickel (Ni), µg/l | 54 | 496 |
| Cadmium (Cd), µg/l | 9 | 56 |
| Lead (Pb), µg/l | 103 | 634 |
| Chromium (Cr), µg/l | 26 | 25 |
| Mercury (Hg), µg/l | 53 | 79 |
| Camphor (mg/l) | 95 | 220 |

Table S4: Overview of transitions for proteomic targeted qualitative analysis

| **Peptide** | **Protein** | **Specificity** | **fragment** | **Q1 m/z** | **Q3 m/z** |
| --- | --- | --- | --- | --- | --- |
| ISAVSTVLEDVQAAGISR* | VLTF-4 viral late | Orthopox | y14 | 908.494 | 1445.759 |
|  | transcription factor |  | y13 | 908.494 | 1358.727 |
|  |  |  | y11 | 908.494 | 1158.611 |
|  |  |  | y10 | 908.494 | 1045.527 |
|  |  |  | y9 | 908.494 | 916.485 |
| VLLLTPEVASLR* | A-type inclusion protein | Orthopox | y10 | 655.906 | 1098.652 |
|  |  |  | y8 | 655.906 | 872.484 |
|  |  |  | y7 | 655.906 | 771.436 |
| QDFDNLIGVR | A-type inclusion protein | Orthopox | y8 | 588.804 | 933.515 |
|  |  |  | y6 | 588.804 | 671.420 |
|  |  |  | y4 | 588.804 | 444.293 |
|  |  |  | y3 | 588.804 | 331.209 |
| VNVFETR | Late transcription factor 1 | Poxviridae | y5 | 432.732 | 651.346 |
|  |  |  | y4 | 432.732 | 552.278 |
|  |  |  | y3 | 432.732 | 405.209 |
|  |  |  | y2 | 432.732 | 276.167 |
| LGINYLIDTTSR | core protein 4a of IMV | Orthopox | y9 | 683.372 | 1082.548 |
|  |  |  | y8 | 683.372 | 968.505 |
|  |  |  | y7 | 683.372 | 805.441 |
|  |  |  | y6 | 683.372 | 692.357 |
| YQSLIPR | core protein 4a of IMV | Orthopox | y5 | 438.751 | 585.372 |
|  |  |  | y4 | 438.751 | 498.340 |
|  |  |  | y3 | 438.751 | 385.256 |
| NDDVLFR | IMV surface membrane 14 kDa | Smallpox | y5 | 439.722 | 649.367 |
|  | fusion protein |  | y4 | 439.722 | 534.340 |
|  |  |  | y2 | 439.722 | 322.187 |
| NDDVLFR heavy peptide |  |  | y5 | 444.726 | 544.348 |
|  |  |  | y4 | 444.726 | 445.280 |
|  |  |  | y2 | 444.726 | 332.196 |

*these peptides were also analyzed on 4000QTRAP

Figure S1: Linear regression model for estimation of the age of specimens V563 and V1588 based on the ratio of D-/L- Asp racemization. Twenty specimens with known age (blue dots) were used for calibration, the estimated age of specimens V563 and V1588 is shown in red. The measured values of the D-/L- Asp ratio are listed in Table S2.

Figure S2: Schematic representation of the genomes derived from specimens V563 and V1588

Figure S3: Chronogram of the maximum credibility tree for Variola viruses generated using BEAST based on the central conserved region of their genomes. Blue bars at nodes represent 95% highest probability density intervals. Numbers on nodes indicate the calculated year of the most recent common ancestor of the clades. The posterior probabilities of all clades is >95% except those labelled with a “#” next to the year.

**Material and Methods S1:**

**Mass Spectrometry**

**Sample preparation**

In order to determine the proteome of the two variola virus specimen, heat-inactivated (15 min/95°C), orthopoxvirus-infected cell culture material was used as control for non-variola viral material. African Green Monkey Kidney cells MA1104 had been infected with cowpox virus strains EP-4 lidil and BR VR302, vaccinia virus strain Elstree B5, and camelpox virus strain CP-1. Mock-infected MA104 cells served as negative control. The sample preparation workflow was based on FASP ([Filter aided proteome preparation - FASP)](http://www.rubicon-net.org/data/file/733_EN_filteraidedproteomepreparationfasp.pdf) [32]. 500 mg of wet tissue was transferred on Amicon® Ultra – 10 kDa filters (Millipore, Carrigtwohill, Ireland), washed with 100 mM ammonium bicarbonate, solubilized in 0.1% RapiGest (Waters, Millfro, MA) for 15 min/95°C and subsequently denatured by 8M guanidinium chloride. Samples were reduced with 100mM Tris (2-carboxyethyl) phosphine hydrochloride (TCEP), alkylated with 300 mM iodoacetamide and digested with 2 μg of sequencing grade trypsin (Promega, Madison, WI) overnight at 37°C. Empore™ SPE Cartridges, C18, standard density, bed I.D. 4 mm (Sigma – Aldrich, Schnelldorf, Germany) were used to desalt peptide mixtures before drying to completion in a speed-vac. Before analysis, the samples were resuspended in 30 μL 2% ACN/0.1% FA. All samples were prepared in technical duplicates.

**MS/MS spectral library**

An UltiMate 3000 RSLCnano system controlled by Chromeleon software (Thermo Scientific) was used for chromatography separation. 1 µl of each sample was vacuum dried, reconstructed in loading phase (2% ACN, 0.05% TFA) and loaded onto 1 of each sample was loaded onto a PepMap100 C18, 3 µm, 100 Å, 0.075 × 20 mm trap column (Thermo Scientific) at 5 µL/min for 5 min. Peptides were separated on a PepMap RSLC C18, 2 µm, 100 Å, 0.075 × 150 mm analytical column (Thermo Scientific) by a gradient formed by mobile phase A (0.1% FA) and mobile phase B (80% ACN, 0.1% FA), running from 4 to 34% in 48 min, and from 34 to 55% of mobile phase B in 15 min at a flow rate of 0.3 µl/min at 40 °C. Eluted peptides were electrosprayed into Q-Exactive using a Nanospray Flex source (Thermo Scientific, Bremen, Germany). Positive ion full scan MS spectra (m/z 300-1800) were acquired using a 1×106 AGC target in the Orbitrap at 70 000 resolution (at m/z 200). The lock mass of m/z 445.12003 ([(C2H6SiO)6+H]+) was used for internal calibration. Top 10 precursors of charge state ≥2 and threshold intensity of 1×105 counts were selected for HCD fragmentation, with an exclusion window of 60 s. The isolation window of 2 Da and normalized CE of 27 was used. Each HCD spectrum was acquired at resolution 70 000, with a 105 AGC target and a maximum 100 ms injection time.

HCD MS/MS spectra were searched in Proteome Discoverer software 1.3 (ver.1.3.0.339, Thermo Scientific) using MASCOT (Matrix Science, London, UK). The tryptic specificity was set with maximum 2 missed cleavages. The mass tolerance was set to 10 ppm for precursors and 20 mmu for product ions. No dynamic modification was allowed while Cysteine thiomethylation was set as a fixed modification and methionine oxidation was set as variable modification. Spectra explained with a cut-off score 0.95 were extracted by Skyline [33] software from the MASCOT .dat file and stored in an MS/MS library.

**LC-Selected Reaction Monitoring analysis**

Peptides were tested using the 4000QTRAP system (Applied Biosystems, Foster City, CA) with a Nanospray III source coupled to a Tempo Autosampler (Applied Biosystems, Foster City, CA) and Tempo nano MDLC capLC system (Applied Biosystems, Foster City, CA). Samples were loaded via 5 μl per sample injection directly onto 75 µm/150 mm column Silicatip (New Objective, Woburn, MA) home filled with reverse phase C18 Spherical silica particle size 3 µm/pore size 100Å. Peptides were separated at a flow rate of 360 nl/min where solvent A was 95% H2O/5% ACN and solvent B was 95% ACN/5% H2O. An 18 min two-stage linear gradient was used where in stage 1 solvent B went from 5% to 40% over 18 min and in stage 2 solvent B went from 40% to 90% over 2 min. Total run length was 60 min per sample. The QTRAP mass spectrometer was operated in SRM mode with Q1/Q3 resolution settings of low/unit. The ion source gas I (GSI), gas II (GSII), curtain gas (CUR), and Interface Heater Temperature were set at 16, 0, 12, and 150°C, respectively. Dwell times were fixed at 5 msec and Total Scan Time (including pauses) was 0,1901 sec. Raw data were acquired using Analyst 1.5.1 (Applied Biosystems, Foster City, CA) and were imported into Skyline in *.wiff format. Retention times as well boundarizations of all peaks were manually inspected and corrected.

In addition, peptides were tested for detectability on a 5500 QTrap mass spectrometer equipped with a nano-electrospray ion source (ABSciex, Concord, Canada). On-line chromatographic separation of the peptides was achieved with an Eksigent Nano liquid chromatography 425 system (Eksigent/ABSciex, Concord, Canada) equipped with a 10 cm fused silica column with 75-μm inner diameter (New Objective, Woburn, USA), packed in-house with ProntoSIL C18 AQ 3-μm 120 Å beads (Bischoff Chromatography, Leonberg, Germany). 1 µl of the peptide mixture corresponding to 1 µg and 0.5 fmol of heavy labeled peptide was loaded from an AS3 autosampler (Eksigent/ABSciex) cooled to 4 °C and separated with a linear gradient from 5 to 35% acetonitrile in 30 min. SRM analysis was conducted with Q1 and Q3 operated at unit resolution (0.7 m/z half maximum peak width) with a dwell time of 35 ms and a cycle time < 2.0 s. Raw data were acquired using Analyst 1.6.2 (ABSciex) and analyzed with Skyline (version 2.1.0 MacCoss Lab Software, Seattle, USA). To extract SRM coordinates, a heavy-labeled purified isotopically labeled synthetic peptide SpikeTides TQL was purchased from JPT Peptide Technologies (Berlin, Germany).

### Selection of surrogate peptides and transitions

The specificity of peptides was proved by the comparing the candidates with the whole unreviewed UniProt database (ver. 20130213) using homemade script running in BioPearl. The peptide VNVFETR was specific for *Poxviridae*, the remainder peptides were Orthopoxvirus-specific with the exception of peptide NDDVLFR, which was found to be specific for VARV. At the beginning, FASTA sequences were imported into Skyline software (University of Washington, Seattle, WA)[33] and following criteria were applied on peptide selection: digestion – trypsin (cleavage at Arg or Lys unless Pro is the next amino acid); no missed cleavage allowed; potentially ragged ends excluded; peptide length - between 5 and 25 amino acids; the first N-terminal amino acid excluded; structural modifications – carbamidomethylation at Cystein. Only peptides matching the criteria and present in the MS/MS library were considered. At the transition level, the criteria were as follows: precursor charge - 2 and 3; fragment charge – 1; ion types – y and b ions; product ions ranging from (m/z>precursor)-1 to last ion for the most of transitions; fragments within a 0.055 Th window around the precursor m/z were excluded. Both the precursor m/z and ion m/z values were restricted to span a range from 300-1400 for 4000QTRAP and 300-1250 for 5500Qtrap respectively.

Three to five most intense product ions found in the library MS/MS spectrum that comply with the above criteria were tested. The collision energy was calculated using default regression functions for 5500TRAP provided in Skyline software and empirically for 4000Qtrap. Where appropriate, both doubly and triply charged precursors were tested even if MS/MS spectrum for just one precursor was available in the library. However, for the final analyses, only doubly charged candidates were selected. Final SRM transition development of analyzed peptides is shown in Table S4.

**Detectability of peptides in orthopoxviruses samples and in the selected samples.**

All peaks were manually inspected to confirm proper selection. Peptides detected as unambiguous chromatographic peaks formed by overlapping of all selected transition signals that showed high similarity degree with peak intensities in the MS/MS spectra, were considered for final SRM method. For the common Poxvirus/Orthopoxvirus peptides, the method was validated on the panel of orthopoxvirus species and the Retention time of these unambiguously detected peptides was then compared with that in analyzed samples. For Smallpox specific peptide NDDVLFR, stable isotope-labeled standards (SIS) containing 13C6 15N2Lys was purchased (JPT Peptide Technologies, Berlin, Germany). C-terminal tag of SIS peptide was cleaved by tryptic digestion and the peptide was desalted on Empore™ SPE Cartridges, C18, standard density, bed I.D. 4 mm (Sigma – Aldrich, Schnelldorf, Germany). The peptide and his light counterpart were analyzed in SRM mode and serial diltuion ranging from 0.5 to 50 fmol/µl showed linearity (analyzed on 4000QTRAP, data not shown). For 5500QTRAP analysis, peptide was spiked in the quantity corresponding to 0.5 fmol per 1 µl loading.

© 2017 by the authors. Submitted for possible open access publication under the
terms and conditions of the Creative Commons Attribution (CC BY) license (http://creativecommons.org/licenses/by/4.0/).
